# Primary Tubercular Chest Wall Abscess in a Young Immunocompetent Male

**DOI:** 10.1155/2014/357456

**Published:** 2014-09-10

**Authors:** Shweta Sharma, R. K. Mahajan, V. P. Myneedu, B. B. Sharma, Nandini Duggal

**Affiliations:** ^1^Department of Microbiology, Dr. Ram Manohar Lohia Hospital and PGIMER, New Delhi 110001, India; ^2^SAG Grade, Department of Microbiology, LRS, Institute of Tuberculosis and Respiratory Disease, Delhi 110030, India; ^3^Department of Radiology, Dr. Ram Manohar Lohia Hospital and PGIMER, New Delhi, India

## Abstract

Chest wall tuberculosis is a rare entity especially in an immunocompetent patient. Infection may result from direct inoculation of the organisms or hematogenous spread from some underlying pathology. Infected lymph nodes may also transfer the bacilli through lymphatic route. Chest wall tuberculosis may resemble a pyogenic abscess or tumour and entertaining the possibility of tubercular etiology remains a clinical challenge unless there are compelling reasons of suspicion. In tuberculosis endemic countries like India, all the abscesses indolent to routine treatment need investigation to rule out mycobacterial causes. We present here a case of chest wall tuberculosis where infection was localized to skin only and, in the absence of any evidence of specific site, it appears to be a case of primary involvement.

## 1. Introduction

Tuberculosis (TB) is a major public health problem with associated high morbidity and mortality if not treated adequately, especially in the developing countries like India which accounts for one-fourth of the global incident TB cases annually. In 2012, out of the global annual incidence of 8.6 million TB cases, 2.3 million were estimated to have occurred in India [1]. As per the WHO Global Report on tuberculosis in 2013, 20% of all the freshly diagnosed cases in India are extrapulmonary. The report also highlights that of 300,000 cases of MDR in the world, India alone has the burden of 64,000 cases [1, 2]. Chest wall tuberculosis is rare form of extrapulmonary TB and accounts for 1–5% of all musculoskeletal TB, which itself is very rare [3]. Sternum remains the most common site to be involved though rib shafts, costochondral junctions, and vertebral bodies can also be involved. Chest wall TB may result from direct inoculation or hematogenous/lymphatic spread or as an extension of underlying pleurapulmonary disease or infection of bony structures. Diagnosis of chest wall tuberculosis is often arduous since clinical presentation may resemble pyogenic abscess and since MOTT are important causes of skin infections failure to respond to conventional ATT may further complicate the diagnosis. Here we are presenting a case of primary tubercular abscess in the chest wall of a 16-year-old boy where the bacilli appeared to have got directly deposited on the damaged skin from an open case of tuberculosis in the family and evolve into a fully developed abscess.

## 2. Case Report

A 16-year-old male presented to the surgical OPD with a painful swelling in the sternal region. Swelling was pea sized (1 × 1 cm) two months back which gradually increased to the present size of 6 × 7 cm. There was history of intermittent fever and loss of appetite for the last 5-6 weeks. The boy recalled getting the skin over his sternum abraded with a metallic religious locket which he used to wear around his neck. There were no respiratory complaints or past history of tuberculosis. There was family history of pulmonary tuberculosis in his brother, who was put on anti-tubercular therapy (ATT) about two weeks before this boy reported to our institution.

**Figure 1:**
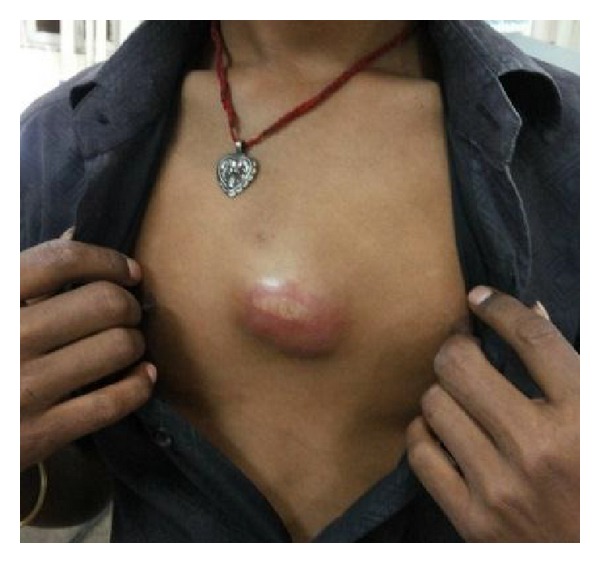
Showing large solitary well defined lesion over sternum.

On examination, the patient was average built, afebrile, and with normal pulse and blood pressure. Respiratory system examination was normal. Local examination revealed a large solitary lesion over sternum of 6 × 7 cm in dimensions. The lesion was soft, fluctuating, tender, warm, with well-defined margins, movable, and not attached to underlying bony structures (Figure 1). There was no involvement of the regional lymph nodes. Since the lesion appeared inflamed, patient was given oral antibiotic—amoxicillin—clavulanic acid combination (Augmentin) for five days. After completion of the antibiotic schedule, when it was observed that the abscess has increased in size, a detailed work up including; incision and drainage of the abscess and CT (computed tomography) chest was planned to assess the extension of the abscess into the surrounding area.

**Figure 2:**
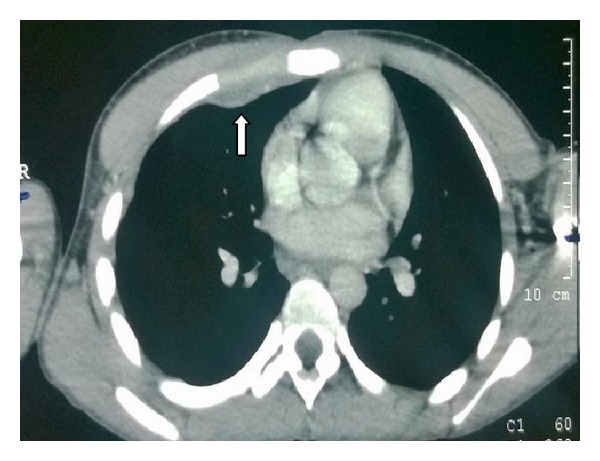
Axial CECT image at lesion level in mediastinal window showing well loculated eliptical hypodense collection with peripheral enhancement on the anterior chest wall with no evidence of mediastinal lymphadenopathy.

**Figure 3:**
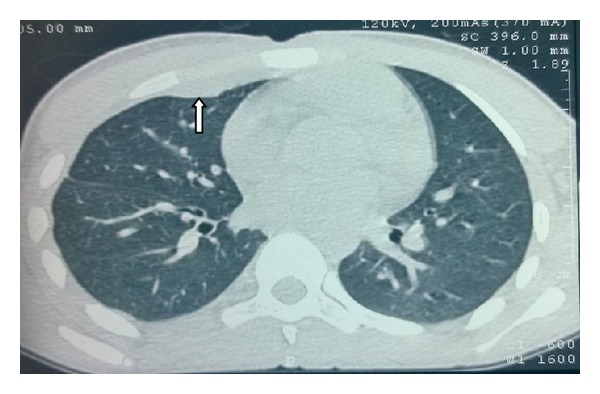
Axial CT image at lesion level in lung window showing no evidence of lung parenchymal involvement.

His haemogram, liver, and renal functions were within reference ranges. Serology for HIV was nonreactive. X-ray chest did not show any abnormality. On the basis of his routine laboratory investigations, incision and drainage (I&D) of the abscess was undertaken on the emergency basis. The drained pus material was sent to the microbiology laboratory for pyogenic culture and Ziehl Neelsen (ZN) staining. There was no growth of any pyogenic organism after 48 hrs of incubation but acid fast bacilli (AFB) were seen in ZN stain. Patient was subjected to CT investigation after I&D had been done. Both plain and contrast enhanced CT (CECT) were done with a proper protocol. Axial contrast enhanced CT in the mediastinal window showed loculated hypodense collection of 1.8 × 1.7 cm in the anterior chest wall in the right parasternal location with peripheral enhancement with no evidence of erosion of ribs or sternum (Figure 2). Also there was no evidence of either lung parenchymal lesion or mediastinal lymphadenopathy (Figure 3). Ultrasound abdomen was within normal limits. Urine sample was negative for AFB. Pus sample was also sent to the LRS Institute of Tuberculosis and Respiratory Diseases (National Reference Laboratory) for culture and sensitivity of* Mycobacterium tuberculosis*. Sample grew* M. tuberculosis *on LJ (Lowenstein Jensen) medium after one week of incubation. In-line probe assay (GenoType MTBDRplus, Hain Life Science), the isolate, was identified as* M. tuberculosis* and found to be sensitive to isoniazid (H) and rifampicin (R) and to other first line drugs, that is, pyrazinamide (Z) and ethambutol (E) by Bactec MGIT (Mycobacteria Growth Indicator Tube) 960 system.

**Figure 4:**
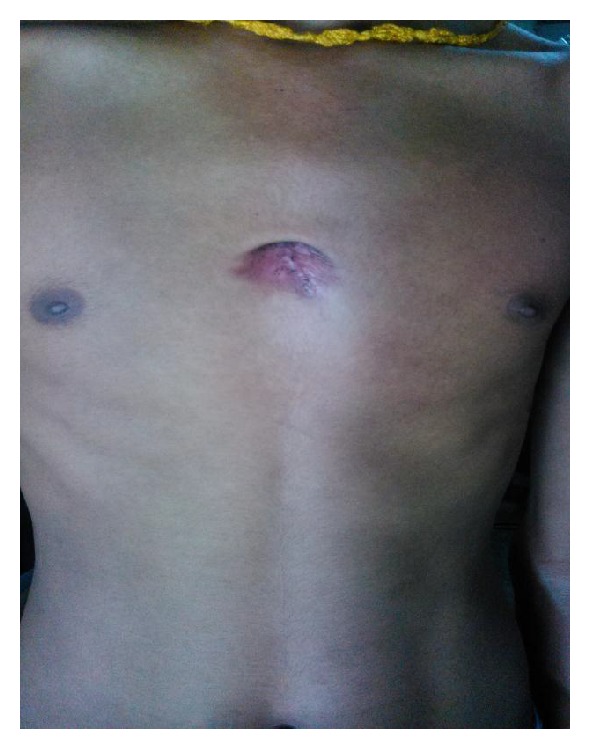
Showing the healed lesion on follow-up examination after 4 months of ATT.

**Figure 5:**
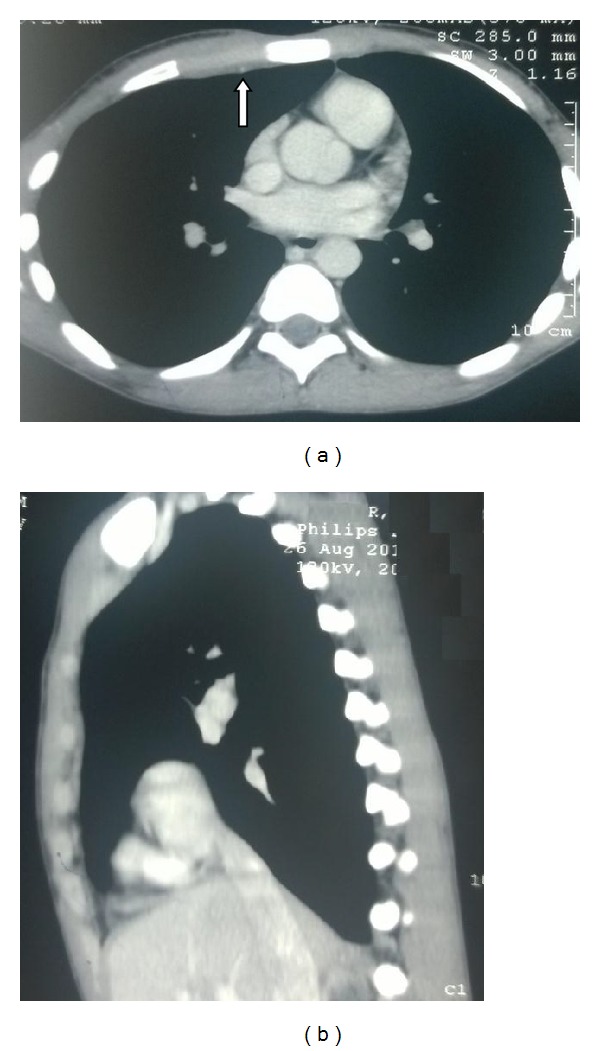
(a) Axial CECT image at lesion level in mediastinal window after 4 months of treatment showing considerable decrease in the collection with no peripheral enhancement. (b) The reconstructed CECT coronal image of the same patient.

The patient was put on Category I treatment (ATT) which consists of an intensive phase of H, R, Z, and E administered under direct supervision thrice weekly on alternate days for 2 months, followed by a continuation phase of H and R thrice weekly on alternate days for 4 months. On follow-up after 4 months, patient responded well to treatment and the abscess resolved drastically (Figures 4 and 5).

## 3. Discussion

Three mechanisms are described in the pathogenesis of chest wall abscess: direct extension from pleural or pulmonary parenchymal disease, hematogenous dissemination of a dormant tuberculous focus, or direct extension from lymphadenitis of the chest wall [4, 5]. Primary tuberculosis of the chest wall is rare and diagnosis in most of the cases is demanding and effortful because the lesions grossly simulate pyogenic abscess or tumour and do not respond to conventional therapeutic interventions. This patient was also prescribed amoxy-clavulanic acid combination for five days but the lesion increased in size though the inflammatory component was relieved. In this particular case, the abscess appeared to be the result of direct inoculation of the organism into the abraded skin because there is history of trauma in the area of lesion with a metallic locket which the boy would wear regularly. The patient comes from low socioeconomic status and used to share bed with his brother, an open case of pulmonary tuberculosis. It is hypothesized that infective droplets from his brother appeared to have settled in the damaged skin to set up infection and develop into an abscess.

Chest wall abscess usually occurs as a solitary lesion, most frequently at the margins of the sternum and in the shafts of the ribs [6]. In the present case, the abscess was present in the upper part of the chest in the sternal region. Chest X-ray of the patient was within normal limits, without any hilar adenopathy. Also the contrast enhanced CT scan suggested the lesion in the subcutaneous and muscle tissue (between the pectoralis muscles) without any involvement of the pleura or underlying bony structures or adjacent lymph nodes. This suggests that the primary focus was neither in the pleura or pulmonary parenchyma, nor in the bony structures and adjacent lymph nodes.

AFB in the aspirated pus signalled the tubercular etiology of the lesion but site of the lesion mandates that the organism is cultured and identified up to the species level because* Mycobacteria* other than tuberculosis (MOTT) are more frequently associated with skin lesions and are one of the significant causes of the treatment failure to ATT [7]. This lesion grew* Mycobacterium tuberculosis* in culture and was susceptible to all the first line drugs in the line probe assay and Bactec MGIT culture and sensitivity system. There is controversy regarding the duration of treatment of chest wall tuberculosis; few reports suggest good response with antitubercular drugs only, while others suggest wide surgical debridement along with antitubercular drugs. However, Revised National Tuberculosis Control Programme (RNTCP) recommends a standard 6-month regimen with 2 months of intensive phase (HRZE) and 4 months of continuation phase (HR) [1]. Patient was started on 1st line drugs for 6 months and on follow-up patient responded well to the treatment and the size of the abscess reduced drastically.

Primary tubercular involvement of extrapulmonary site like chest wall abscess is extremely rare. Demonstration of acid fast bacilli should not form the basis of starting antitubercular treatment; rather the organism requires to be identified up to the species level to exclude the possibility of MOTT as the causative agents which would require separate treatment protocol. In country like India that has massive pool of tuberculosis cases and in the background of priority to pick up open cases, there is a possibility that extrapulmonary tuberculosis may be missed or misdiagnosed. Identification of extrapulmonary isolates would be absolutely essential for instituting right therapeutic intervention but with limited facilities for mycobacterial culture and sensitivity some kinds of linkages are required to provide support services to sites engaged in handling tuberculosis patients.
